# Bioprospecting of probiotics with antimicrobial activities against *Salmonella* Heidelberg and that produce B-complex vitamins as potential supplements in poultry nutrition

**DOI:** 10.1038/s41598-020-64038-9

**Published:** 2020-04-29

**Authors:** Sabrina da Silva Sabo, Maria Anita Mendes, Elias da Silva Araújo, Ligia Bicudo de Almeida Muradian, Edson Naoto Makiyama, Jean Guy LeBlanc, Primavera Borelli, Ricardo Ambrósio Fock, Terezinha Knöbl, Ricardo Pinheiro de Souza Oliveira

**Affiliations:** 1 Department of Biochemical and Pharmaceutical Technology, School of Pharmaceutical Sciences, University of São Paulo, São Paulo, Brazil; 2 Chemical Engineering Department, University of São Paulo, São Paulo, Brazil; 3 Department of Food and Experimental Nutrition, School of Pharmaceutical Science, University of São Paulo, São Paulo, Brazil; 4 Department of Clinical and Toxicological Analysis, School of Pharmaceutical Science, University of São Paulo, São Paulo, Brazil; 5 CERELA-CONICET, San Miguel de Tucumán, Tucumán, Argentina; 6 Department of Pathology, School of Veterinary Medicine and Animal Science, São Paulo, Brazil, University of São Paulo, São Paulo, Brazil

**Keywords:** Antimicrobial resistance, Applied microbiology

## Abstract

The demand for animal protein for human consumption has been risen exponentially. Modern animal production practices are associated with the regular use of antibiotics, potentially increasing the emerging multi-resistant bacteria, which may have a negative impact on public health. In poultry production, substances capable of maximizing the animals’ performance and displaying an antimicrobial activity against pathogens are very well desirable features. Probiotic can be an efficient solution for such a task. In the present work, lactic acid bacteria (LAB) were isolated from chicken cecum and screened for their antagonistic effect towards many pathogens. Their capacity of producing the B-complex vitamins folate and riboflavin were also evaluated. From 314 isolates, three (C43, C175 and C195) produced Bacteriocin-Like Inhibitory Substances (BLIS) against *Staphylococcus aureus* (inhibition zones of 18.9, 21.5, 19.5 mm, respectively) and also inhibited the growth of *Salmonella* Heidelberg. The isolate C43 was identified as *Enterococcus faecium*, while C173 and C195 were both identified as *Lactococcus lactis* subsp. *lactis*. Moreover, the isolates *L. lactis* subsp. *lactis* strains C173 and C195 demonstrated high potential to be used as probiotic in poultry feed, in addition to their advantage of producing folate (58.0 and 595.5 ng/mL, respectively) and riboflavin (223.3 and 175.0 ng/mL, respectively).

## Introduction

The use of antibiotics in animal production originated more than 70 years ago, when chlortetracycline was shown to improve the growth and health of chicks^1^. Since then, antibiotics have not only been widely used in the treatment and prevention of diseases, but also to improve animal growth and productivity^2^. Their use in farming animals to increase profits and their correlation with antimicrobial resistance in humans is a polemic subject since some antibiotics used in animals are the same or are closely related to the antibiotics used in human therapeutics^3^.

Even though the use of antibiotics in veterinary medicine is used cautiously and is well controlled by national and international organizations, many studies have recognized that using antibiotics in farming animals may have a negative impact on public health^4^. This impact may be related to the selection of resistant microorganisms, zoonotic transmission or transference of resistance genes between animal and human bacteria^5^.

The first evidence of this correlation was reported in 1995, when after banning avoparcin, which is chemically related to the glycopeptide vancomycin commonly used as a growth promoter for broilers, there was a reduction in the prevalence of vancomycin-resistant enterococci (VRE) from 72% in 1995 to 2% in 2005^6^. An equivalent decline in prevalence of *E. faecium* resistant to avilamycin was observed after the prohibition of this antibiotic^6^.

In Denmark, the prevalence of VRE in pigs persisted for three years after the ban of avoparcin, until the growth promoter tylosin (a macrolide) was also prohibited. The genetic characterization of VRE isolated from these animals revealed the presence of plasmids encoding resistance to glycopeptides and macrolides, suggesting that the resistance to vancomycin could have been a consequence of using tylosin after the avoparcin prohibition^7^.

Brazil is the second largest producer and largest exporter of poultry meat in the world, with poultry products traded in more than 150 countries. Data from the Brazilian Association of Animal Protein, for the year 2017, recorded the production of 13,056 million tons of chicken meat, with exportation of 4,320 million tons^8^.

*Salmonella* Heidelberg is one of the emerging serogroups in Brazil and there is a concern in the poultry sector because this pathogenic microorganism is multiresistant and carries molecular determinants of beta-lactam resistance^9^. For many decades, the Poultry Health Program has used strategies focused on controlling *Salmonella* Gallinarum, *Salmonella* Pullorum, *Salmonella* Typhimurium and *Salmonella* Enteritidis^10^. The prevalence of these serovars has been well controlled, but there is a difficulty in controlling the avian paratyphoid, especially in Heidelberg and Minesota serovars.

Recently, in Brazil, it has been identified between a 5% and 11% positive result for non-typhoidal *Salmonella* in avian origin samples, where *Salmonella* Heidelberg and *S*. Typhimurium have been the most common^11–14^. Moreover, in United States, *S*. Heidelberg is currently the 12^th^ most common serovar of *Salmonella enterica* causing salmonellosis and results in twice the average incidence of blood infections caused by non-typhoidal salmonellae. Several outbreaks of salmonellosis caused by *S*. Heidelberg resulted from the poultry processing^15^.

Increasing evidences led the World Health Organization suggest the phase out or even the complete ban of antibiotic growth promoters (AGPs)^16–18^. The European Commission banned the marketing and use of AGPs in feed nutrition since 2006^19^. In the United States, the use of fluoroquinolones in poultry was banned in 2005. After that, the number of fluoroquinolone-resistant *Campylobacter* infections in humans has significantly decreased^20^. Such phasing-out of AGPs increased the pressure of the livestock industry to search for viable alternatives that can improve the natural defense mechanism of these animals^21,22^. In this sense, the use of probiotic microorganisms has gained much attention.

Probiotics are defined as: “living microorganisms which, when administered in adequate quantities, confer benefits to the host’s health”^23^. When used as a feed additive these microorganisms are associated with maintaining a balanced intestinal microbiota, increasing the resistance against pathogenic bacteria and improving the immunity of the host^24^. Such benefits by probiotic strains can be due to their capability of producing fatty acids and organic acids, as well as the synthesis of compounds such as vitamins and antimicrobial biomolecules like bacteriocins, also named as bacteriocin-like inhibitory substance (BLIS), which act specifically against certain pathogens or in the activity of certain enzymes^23^. The BLIS nomenclature was proposed by Tagg, Dajani and Wannamaker^25^ and is recommended when a newly discovered bacteriocin is not completely characterized regarding its amino acid or nucleotide sequences.

Probiotic strains should not compete with the microorganisms of the normal microbiota, instead they should interact in symbiosis. Moreover, they must be resistant to bile and acids from the gastrointestinal tract and have the ability to adhere to the intestinal mucosa cells, rapidly multiplying in order to always be present in the intestinal lumen and not be completely eliminated during intestinal peristalsis. Another fundamental criterion is the safety for use in humans or animals^26^. Probiotic microorganisms usually used in food, pharmaceutical and veterinary industries belong to the Lactic Acid Bacteria (LAB) group since these have being historically used in the manufacture of dairy foods and as starter cultures and have received the “generally regarded as safe” (GRAS) status from the US Food and Drug Administration^27^.

It should be emphasized that during animal management, several stressful situations can affect their growth and health^28^. Although breeders provide balanced diets, clinical or subclinical deficiencies of some essential vitamins for proper animal development may occur, which can significantly affect production costs^29^. Although LAB are usually known as auxotrophic for some vitamins, certain strains have the ability to synthesize water soluble vitamins, including those comprising the B group^30^. In poultry nutrition, B-complex vitamins such as folates and riboflavin are essential as evidenced by diverse symptoms when they are deficient^31^.

As mentioned above, the production of antimicrobial substances by probiotic strains, such as bacteriocins, makes the use of these microorganisms additionally advantageous. Although several microorganisms can produce bacteriocins, those produced by LAB have gained importance due to their potential use as biopreservatives for foods^32^. By definition, bacteriocins are peptides or proteins synthesized via the ribosome and exported to the extracellular medium which, in turn, have antimicrobial activity against other closely related bacterial species^33^. Inhibition of pathogenic bacteria by LAB is mediated mainly by the production of these biomolecules, which stimulate animal productivity, providing weight gain, strengthening the immune system and reducing the mortality rate by controlling pathogens that commonly affect pigs, cattle and birds^34–37^.

In poultry production, one of the main goals is the reduction, through the introduction of probiotic bacteria in their diets, of the foodborne pathogens *Salmonella* ssp. and *Campylobacter* ssp^38,39^. These pathogenic microorganisms are the major cause of diseases in humans transmitted by contaminated foods, with birds being an important reservoir, when contaminated by such pathogen^39,40^.

Based on all of the above, the aim of this study was to isolate potential probiotic LAB from chicken cecum content capable of producing antimicrobial activity against *Salmonella* Heidelberg, and produce the B-complex vitamins riboflavin and folate.

## Materials and methods

### Sampling of chicken cecum and LAB isolation

The screening for interesting LAB was conducted based on the protocol described by Messaoudi *et al*.^41^. Broiler chickens (*Gallus gallus domesticus*) in our study were fed with animal protein and AGP-free commercial feed and water *ad libitum*. They were maintained under controlled management conditions (diet, room temperature, cleaning). When animals reached 12-weeks of age, they were sacrificed with an anesthetic overdose (barbiturate). The cecum was carefully removed from the carcasses under aseptic conditions and transported to the laboratory in a refrigerated thermal box (~4 °C). Inside a laminar airflow biological cabinet, 10 g of it was transferred to a sterile plastic bag containing 90 mL of 0.1% (w/v) bacteriological peptone water (Sigma-Aldrich, Missouri, USA). It was homogenized for 2 min in a Stomacher device (SPlabor, São Paulo, Brazil), and the resulting product was serially diluted in sterile 0.85% (w/v) NaCl solution. One hundred μL of each dilution was transferred to Petri dishes (90 × 15 mm) containing Man, Rogosa and Sharpe (MRS) agar medium (Difco, Michigan, USA), Bifidus Selective Medium (BSM) agar (Sigma-Aldrich), and M17 agar medium (Difco, Michigan, USA) supplemented with 0.1 g/L of cycloheximide (Inlab, São Paulo, Brazil), as selective culture media for *Lactobacillus*, *Bifidobacterium*, *Lactococcus* genus, respectively. Using a sterile Drigalski handle, the dilutions were spread over the surface of the agar medium plates and, anaerobically incubated for 48 h at 30, 37, or 45 °C in a microaerophilic jar using a GasPak EZ Container System (BD Diagnostic Systems, Maryland, USA) to ensure hypoxia. After this period 314 colonies showing different morphologies were randomly selected and subcultured onto the aforementioned agar media plates, which were used in the following steps.

This study The study was approved by Ethics Committee on the use of animals of School of Veterinary Medicine and Animal Science in the University of São Paulo (registration number 7843030717). All experiments were performed in accordance with relevant guidelines and regulations.

### Screening for LAB with antimicrobial activity against S. Heidelberg

*S*. Heidelberg strains IOC 969/17, isolated from chicken carcass sample was provided by FioCruz (Rio de Janeiro, RJ, Brazil) and used as bioindicator strain to evaluate the antimicrobial activity of the 314 isolates. The bioindicator strain was cultured in Brain Heart Infusion (BHI; Difco, Michigan, USA) broth for 18 h at 37 °C and 100-fold diluted (to approximately 10^6^ CFU/mL). This suspension was used to inoculate BHI soft agar (0.75% [w/v] of agar) at 1/10 ratio (v/v) and the resulting mixture was used to overlay the agar media plates containing the 314 isolates. The plates were incubated at 37 °C for 24 h. Isolates showing the largest inhibition zones against *S*. Heidelberg IOC 969/17 were selected and subcultured in the appropriated medium (BHI, M17 or BSM) and temperature (30, 37 or 42 °C), according to the isolates preferences. They were first characterized morphologically by microscopy, Gram staining, and the detection of catalase activity. Gram-positive, catalase negative isolates were considered as potential LAB and used in further studies. All the isolates were stored at −70 °C using 20% (v/v) glycerol as cryopreservative.

### Evaluating the nature of the antimicrobial activity

To evaluation of the nature of the antimicrobial activity demonstrated by the selected isolates against *S*. Heidelberg IOC 969/17 was performed using previously established protocols^42^. The isolates were cultured in their preferential media broth and temperature for 24 h, then centrifuged (Boeco, Hamburg, Germany) at 12,000 *g* at 4 °C for 15 min. From each culture broth, the supernatant was recovered and the pH adjusted to 6.0 – 6.5 with 1.0 M NaOH. The samples were filtered through 0.22-μm hydrophilic PVDF membranes (Millipore, Maryland, USA), resulting in cell-free supernatants labeled as CFS-A. Then, these CFS-A samples were investigated regarding their antimicrobial effect against *S*. Heidelberg IOC 969/17.

The potential inhibitory effect of CFS-A samples against *S*. Heidelberg IOC 969/17 was investigated by the agar diffusion method, specifically spot-on-the-lawn methodology^42^. For that, the bioindicator strain was precultivated at 37 °C in BHI broth and after 18 h it was 100-fold diluted (approximately 10^6^ CFU/mL) and 1 mL of this suspension was transferred to a Petri dish (90 × 15 mm) containing 9 mL of BHI melted soft agar (supplemented with 0.75% [w/v] of agar). After solidification, 20 μL of each CFS-A was pipetted onto the agar surface and, finally, the plates were incubated at 37 °C for 24 h. The inhibition zone formed after such period by the CFS-A was measured using a digital caliper and the antimicrobial activity was associated with BLIS production.

Additionally, attempting to verify the inhibitory spectrum caused by BLIS present in the CFS-A, the samples were also tested against other bioindicator strains, such as *Listeria monocytogenes* CECT-934, *Salmonella enterica* CECT-724, *S. aureus* CECT-237, *Escherichia coli* ATCC 25922, *Carnobacterium piscicola* CECT-4020, *Listeria innocua* CLIST 2052. All strains were cultured under conditions similar to *S*. Heidelberg IOC 969/17, except for *C. piscicola* CECT-4020, which was cultured in MRS broth. The antimicrobial activity was determined in technical triplicate.

To see if BLIS were of proteinic nature CFS-A samples which exhibited inhibition zones against the bioindicator strains were treated with 3 mg/mL (w/v) of the proteolytic enzyme protease XIV (Sigma-Aldrich, Missouri, USA) which was added to 1 mL of each CFS-A and incubated at 30 °C for 2 h^42^. After that, the solutions were evaluated regarding their remaining antimicrobial activity against *S. aureus* CECT-237, following the protocol described above. The solutions demonstrating no inhibition zones were considered, in this study, as being BLIS.

The isolates producing CFS-A with antimicrobial activity against most bioindicator strains were cultured and after 24 h incubation the supernatants were once again recovered. At this time, the aim was to investigate the antimicrobial activity of the organic acids supposedly produced against *S*. Heidelberg IOC 969/17, so no pH adjustment were performed, resulting in CFS-B samples. To carry out this assay, the bioindicator strain was cultured in 10 mL of BHI broth at 37 °C for 24 h and diluted in 2× BHI broth to achieve a cell concentration of 0.2 optical density (OD) units at 600 nm per milliliter. After that, 100 μL of this solution was placed into wells of a 96-well sterile covered microplates (TPP, Trasadingen, Switzerland) together with 100 μL of each CFS-B sample from the selected isolates Finally, the microplate was covered and incubated on a microplate reader (Bioteck Instruments, Vermont, USA) at 37 °C, where OD was recorded every 1 h at 600 nm for 24 h.

### Identification of LAB producing B-complex vitamins

The isolates, which displayed the largest inhibition zones against most of bioindicator strain, were selected to evaluate their ability to produce the B-complex vitamins riboflavin and folate. The protocol was followed according to Laiño *et al*.^43^. Briefly, the selected isolates, stored at −70 °C, were first activated in the above-mentioned medium and conditions, according to the isolates preferences. After 24 h incubation, 1 mL-aliquots were centrifugated at 12,000 *g* at 4 °C for 15 min. The supernatants were discarded and the pellet cells were washed 3 times with sterile 0.85% (w/v) saline, being resuspended at the original culture volume after this 3-washes steps. The resulted solutions were used to inoculate at 2% (v/v) the Folic Acid Casei Medium (FACM; Himedia, Mumbai, India), and the Riboflavin Assay Medium (RAM; Difco, Maryland, USA), which are free from folates and riboflavin, respectively. The cultures were incubated without agitation at 37 °C for 18 h and after growth those 3-washes and resuspending step was repeated. The resulting solutions were used to inoculate at 2% (v/v) fresh FACM or RAM. In order to deplete the reserves of folates or riboflavin from the isolates, this last step was repeated 7 times with the cultures showing good growth (observed by increased turbidity). The isolates which did not grow in FACM or RAM were not used in further studies.

#### Folate quantification

A 500 μL sample of isolates grown in FACM were taken and diluted with same volume of protecting buffer (0.1 mol/L phosphate buffer, pH 6.8, containing 1.5% [w/v] ascorbic acid to prevent vitamin oxidation and degradation) followed by immediate centrifugation at 12,000 *g* at 4 °C for 5 min. The obtained supernatants were then boiled (100 °C) for 5 min and stored at –70 °C until folate quantification.

Folate quantification was determined by a microbiological assay using *Lactobacillus casei* subsp. *rhamnosus* ATCC 7469, provided by Fiocruz (Rio de Janeiro, RJ, Brazil) as the indicator strain according to Horne and Petterson^44^. The strain was previously cultured in MRS broth at 37 °C for 24 h and cryopreserved using 20% (v/v) glycerol and stored at –70 °C. Fresh MRS broth was used to activate the strain under the same mentioned conditions. After growth, 1mL-aliquot was washed 3 times with sterile 0.85% (w/v) saline, resuspended in the original volume, and used to inoculate at 2% (v/v) fresh FACM, which was incubated for 24 h at 37 °C. This last step was repeated and the second culture was used to perform the folate determination, as follows bellow.

The frozen samples were thawed at room temperature (24 °C) and processed in light-reduced conditions^45^. The samples were then diluted with protection buffer and 100 μL of each sample was placed into wells of a 96-well sterile covered microplates (TPP, Trasadingen, Switzerland). The folate concentration of each diluted sample was determined in triplicate. One hundred microliters of the indicator strain *L. casei* subsp. *rhamnosus* ATCC 7469, grown in FACM as described above, was added to each well and mixed. The growth in the presence of the samples was compared to those with a standard curve prepared using HPLC grade folic acid (Sigma-Aldrich, Missouri, USA) diluted in the protection buffer at different concentrations (between 0 and 0.1 ng/mL).

#### Riboflavin determination

The determination of riboflavin was carried out according to de Arruda *et al*.^46^ with some adaptations. Briefly, the selected isolates were cultured following above-described conditions. After 24 h incubation, using a 100 mL-Erlenmeyer flasks, 5 mL of each culture broths were added with 45 mL of 0.1 M hydrochloric acid. The resulting solutions were heated in a shaking thermoregulated bath at 100 °C/100 rpm for 30 min. The solutions were cooled to room temperature (24 °C) and had their pH adjusted to 4.6 using 2.5 M sodium acetate. After adding 0.5 g of the enzyme diastase from *Aspergillus oryzae* (Merck Millipore, Massachusetts, USA), the solutions were incubated for 2 h at 42 °C in a thermoregulated bath. After such enzymatic treatment and cooling at room temperature (24 °C), the solutions were transferred to 100 mL-volumetric flasks and the volumes were completed with deionized water, homogenized and filtered through 0.45-μm hydrophilic PVDF membranes (Nova Analítica, São Paulo, Brazil) for injection into the chromatographic system. This step was carried out in triplicate.

The chromatography system was equipped with two Shimadzu (LC-20AT) pumps, Shimadzu (SIL-20A Autosampler) autosampler; Shimadzu (RF-10AXL) Fluorescence detector; Software LC-Solution, Shimadzu CBM-20A (SCL-10AVP) system. The method to quantify riboflavin concentration of each sample was according to de Arruda *et al*.^46^. Twenty μL of samples solutions was injected into column C18 reversed-phase (RP-18 spherical 5 mm/250 mm 4.6 Shimadzu1 Shim-pack, P/N 228- 34937-92) with pre-column (5 mm/10 mm 4.6 mm Shimadzu1 Shim-pack, VP-ODS P/N 228-34938-91). The mobile phase was composed of phosphate buffer pH 7.2 (10 mM KH_2_PO_4_·3H_2_O) and dimethylformamide (85:15); flow: 1 mL/min; detected by fluorescence: Ex 450 nm; Em 530 nm.

### Identification of the selected LAB isolates using matrix-assisted laser desorption/ionization time-of-flight mass spectrometry (MALDI-TOFMS)

To identify the selected isolates, MALDI-TOFMS analysis were adopted according to Alves *et al*.^47^. They were cultured in plates containing MRS or BSM 1.5% agar and after 24 h incubation under anaerobic condition, the isolates were transferred from the plates to a 1.5 mL-microtube containing 200 μL of distilled water and homogenized during 1 min in a vortex device. Then, 900 μL of ethanol was added and the samples were centrifuged at 12,000 *g* at 4 °C for 5 min. The supernatant was discharged, and the residual alcohol was left to dry at room temperature. The resulting pellet was suspended in 50 μL of 70% formic acid and 50 μL of acetonitrile was added, and the sample was homogenized using a vortex. An α-cyano-4-hydroxycinnamic acid matrix was prepared as a saturated solution in 50% acetonitrile and 2.5% trifluoroacetic acid. Subsequently, 1 μL of samples previously treated and 1 μL of matrix solution was spotted onto a steel target plate and allowed to dry at room temperature.

The mass spectrometry analyses were conducted using an UltrafleXtreme MALDI-TOF mass spectrometer (Bruker Daltonics, Germany) operating in the linear positive ion mode. Mass spectra were acquired in a mass range from 2 to 20 kDa with ions formed by irradiation of smart beam using a frequency of 2000 Hz, PIE 100 ns, 7 kV lens. The voltages for the first and second ion sources were 25 kV and 23 kV, respectively. Bacteria were identified by means of the Biotyper 3.1 database. The identification cut-off values higher than 2 and 1.7 were used for species and genus identification, respectively.

### *In vitro* evaluation of desirable criteria to consider the selected isolates as probiotic LAB

#### Tolerance to low pH

To verify the isolates’ tolerance to low pH conditions, it was adopted the protocol developed by Zoumpopoulou *et al*.^48^ and Argyri *et al*.^49^. Briefly, the isolates were cultured in the preferential medium and temperature. After 18 h incubation, the culture broths were centrifugated at 4,470 *g* at 4 °C for 10 min and the pellets cells were washed twice with phosphate buffered saline (PBS) (pH 7.2) before being resuspended in PBS solution adjusted to pH 2.5. To reflect the time spent by food in the stomach, the resulting solutions were incubated at 37 °C for 0, 0.5, 1.0, 2.0 and 3.0 h and the tolerance was observed in triplicate in terms of viable colony counts and enumerated on the same medium initially used to cultivate the isolates added with 1.5% (w/v) agar.

#### Tolerance to bile salts

The tolerance to bile salts was evaluated adapting the protocol followed by Maragkoudakis *et al*.^50^ and Pedersen *et al*.^51^. The isolates were cultured in MRS broth at 37 °C for 24 h. After that, 1 mL of such cultures broths were used to inoculate, individually, 9 mL of MRS broth supplemented with 0.5% (w/v) porcine bile extract (Sigma-Aldrich, Missouri, USA). The resulting solutions were incubated at 37 °C for 4 h. The isolates demonstrating bile resistance were assessed in terms of viable colony counts, enumerated after incubation for 0, 1, 2, 3 and 4 h, which the latter being representative of the time spent by food in the small intestine.

#### Hemolytic activity

In order to investigate if the isolates produced hemolytic activity, they were cultured in the appropriated conditions mentioned before and after 24 h incubation, the cultures were streaked onto Mueller Hinton agar surface (Difco, Michigan, USA) previously supplemented with 5% (v/v) sheep blood (Ebefarma, Rio de Janeiro, Brazil). The plates were incubated at 37 °C under anaerobic condition and after 24 h, they were exanimated for signs of β-hemolysis (clear zones around colonies), α-hemolysis (green-hued zones around colonies) or γ-hemolysis (no zones around colonies)^49^.

#### Antibiotic susceptibility

Antibiotic susceptibility was determined by the modified Kirby-Bauer disc diffusion method^52^. Discs (all provided by Laborclin, São Paulo, Brazil) of the following antibiotics were used: ampicillin (10 μg); chloramphenicol (30 μg); clindamycin (2 μg); gentamicin (10 μg); streptomycin (10 μg); rifampicin (5 μg), and vancomycin (30 μg), all selected following the suggestion stated on the document *Opinion of the Scientific Committee on Animal Nutrition on the criteria for assessing the safety of micro-organisms resistant to antibiotics of human clinical and veterinary importance*^53^. Briefly, the isolates were cultured in the above-mentioned medium and conditions. After 24 h, the concentration of isolates’ cell suspension was adjusted according to 0.5 McFarland (Probac, São Paulo, Brazil) standard and spread over the surface of Mueller Hinton agar plates. When the suspensions were dried, the discs were carefully placed onto the plates and anaerobically incubated at 37 °C for 24 h. Presence or absence of inhibition zones was defined as sensitivity or resistance, respectively, interpreted according to the cut-off levels proposed by Charteris *et al*.^54^.

#### Coexistence test

The compatibility among the selected isolated was performed according to Guo *et al*.^55^. The selected isolates were cultured in the appropriate conditions and after 24 h they were streaked perpendicularly and across each other on MRS 1.5% (w/v) agar plates. The plates were anaerobically incubated at 37 °C and after 24 h antagonism or absence of was observed.

#### Cell surface hydrophobicity

The cell surface hydrophobicity assay was conducted according to García-Hernández *et al*.^56^. In this sense, the hydrophobicity was determined as the ability of the isolates to adhere to hydrocarbons (MATS: Microbial Adhesion to Solvents), using toluene as solvent. This assay was carried out culturing the isolates in appropriated medium and temperature for 24 h. A 3 mL-aliquot from each culture broth were centrifugated at 4.470 *g* at 4 °C for 10 min and the pellets were washed twice with PBS (pH 7.2) and resuspended in same buffer until achieve the concentration of 1.0 OD at 560 nm (A_b0_). To three milliliter of these solutions, 0.6 mL of toluene (Synth, São Paulo, Brazil) was added and gently mixed for 2 min. After an incubation period of 1 h at 37 °C, the aqueous phase (bottom phase) was carefully collected and the OD_560_ nm was determined again (A_b1_). Percentage of MATS was calculated using the following equation:

1 $$ \% MATS=\frac{({A}_{b0}-{A}_{b1})}{{A}_{b0}}\times 100$$

Isolates with MATS above 50% were considered as hydrophobic.

#### Bacterial adhesion to intestinal epithelial cells

The adhesion capacity of the isolates was investigated according to Jensen *et al*.^57^ with some modifications. Basically, 2.10^5^ cells from human colon adenocarcinoma (Caco-2; ATCC HTB-37, Manassas VA, USA) were sub-cultivated in 24-well culture plates containing Dulbecco’s modified Eagle’s medium (DMEM; Vitrocell Embriolife, Campinas, Brazil) containing low glucose, and 20% (v/v) fetal bovine serum (Vitrocell Embriolife, Campinas, Brazil) and 100 U/ml penicillin/streptomycin (Sigma-Aldrich, St. Louis, USA). Then, the 24-well plates were incubated at 37 °C in a humidified atmosphere of 5% CO_2_ and 95% air until reach confluence growth (3 days). The assays were carried out, by culturing the bacterial isolates on MRS broth for 24 h at 37 °C, centrifugation (10.000 *g* for 10 min) and the resulting pellets were resuspended in DMSO without antibiotics supplementation. Before experiments, the Caco-2 monolayers were washed twice with PBS to remove antibiotics from the original cell media and 1 mL of each bacterial cells solution (10^7^ CFU/mL) were individually added to the wells. Subsequently, the well-plates were incubated at 37 °C for 1, 2 or 4 h in order to determine the best adhesion period. After that, the bacterial solutions were removed from each well and the monolayers were washed twice with PSB to remove non-adherent bacteria. Finally, the monolayers were lysed by addition of PBS added with 0.1% Triton-X100 (Sigma-Aldrich, St. Louis, USA). The resulting suspension with viable adhered bacteria were serially diluted and plated onto MRS agar by pour plate method. After 48 h incubation in anaerobic jar, the number of colony forming units per mL was counted. Bacterial adhesion capacity was expressed as a percentage, which was calculated using the ratio of the number of bacterial cells that remained attached in the monolayer to the total number of bacterial cells added initially to each well. The experiment was performed in biological triplicate.

### Statistical analyses

The results were expressed as the mean and standard deviation (S.D.). The counts of viable bacteria were transformed to log values. The values in the tolerance test were compared by Student’s t-test. P-values less than 0.05 were regarded as a significant difference.

## Results and discussion

### Evaluation of antimicrobial activities in LAB isolates

**Figure 1:**
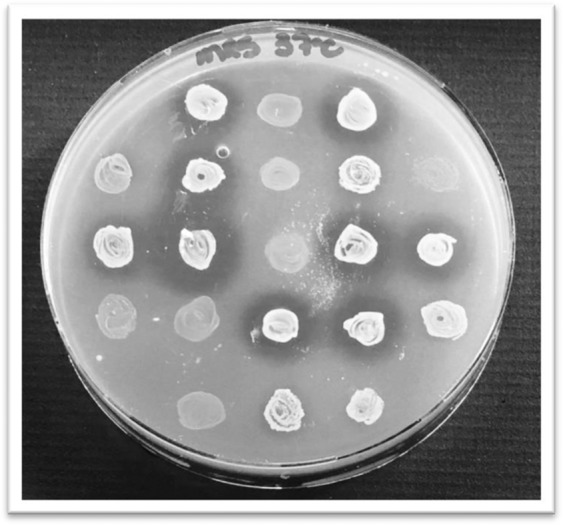
Inhibition zones against *Salmonella* Heidelberg IOC 969/17 produced by LAB colonies isolated from chicken cecum content.

From the cecum content of broiler chickens, 314 colonies were aleatory selected and their antimicrobial activity against *S*. Heidelberg IOC 969/17 were evaluated. From these assays 35 isolates stood out compared to the others, producing large inhibition zones against the bioindicator strain (Fig. 1) and these were characterized as being Gram-positive with coccus- and rod-shaped, non-sporulating and catalase-negative, which are predominant features of the LAB group^58^.

The antimicrobial activity of pH neutralized samples (CFS-A) was evaluated against *S*. Heidelberg IOC 969/17 and did not present any antimicrobial activity. Considering this result and the fact that BLIS antimicrobial activity is target-specific^59^, the samples were tested against other pathogens including *L. monocytogenes* CECT-934, *S. enterica* CECT-724, *S. aureus* CECT-237, *E. coli* ATCC 25922, *C. piscicola* CECT-4020 and *L. innocua* CLIST 2052.

Interestingly, the highest antimicrobial activities were observed against Gram-positive species, especially *S. aureus* CECT-237 (see Table 1). None of the neutralized samples were capable of producing inhibition zones against *S. enterica* strain CECT-724 or *E. coli* ATCC 25922, both being Gram-negative. According to Prudêncio *et al*.^60^, bacteriocins produced by Gram-positive microorganisms are not effective against Gram-negative ones since the latter are resistant to their action. This occurs because Gram-negative bacteria have are very complex outer membrane that make them resistant to certain antibiotics, dyes, and detergents^61^. Certain treatments can alter this unique outer membrane and make it permeable and, consequently, making the bacterial cells sensitive to bacteriocins^62^. Particularly, *Salmonella* species have been frequently pre-exposed to chelating agents, for example EDTA which acts by destabilizing the complex membrane of Gram-negative bacteria and cause the release its structural components or disrupt the lipopolysaccharide layer (LPS), allowing the bacteriocin to act^60^. In this sense, this strategy could be a useful alternative to overcome the *Salmonella*’s resistance to the BLIS produced by our isolates, being therefore a test that will be carried out in the future. Based on the results presented in Table 1, the isolates labeled as C43, C173 and C195 were chosen for further study.

**Table 1:** Antimicrobial activity of CFS-A extracted from the culture of the isolates against bioindicator strains.

Name of isolates	*L. monocytogenes* CECT 934	*S. aureus* CECT 237	*C. piscicola* CECT 4020	*L. innocua* CLIST 2052
	Inhibition zones (mm)			
C12	—	13.8	—	—
C13	13.0	17.6	—	12.3
C14	15.9	17.7	23.1	12.3
C15	12.6	8.7	15.5	12.4
C18	11.9	15.9	16.2	11.9
C19	12.5	14.8	—	13.1
C22	11.2	—	—	11.0
C23	11.1	16.9	22.4	12.1
C25	—	15.5	—	—
C26	10.8	—	—	10.6
C28	12.6	16.3	15.1	12.9
C34	—	—	—	—
C35	—	14.2	—	—
C43	12.5	18.9	23.3	14.1
C45	11.4	16.2	—	11.6
C52	10.9	15.7	—	11.8
C78	—	14.9	—	—
C79	10.3	—	—	11.7
C81	12.8	18.3	24.3	—
C83	16.0	17.9	23.4	12.8
C127	—	—	—	—
C129	—	—	—	—
C138	—	13.8	—	—
C147	—	15.8	—	—
C149	—	13.8	—	—
C150	—	14.3	—	10.2
C154	—	16.5	—	10.7
C155	10.6	—	—	10.9
C165	—	—	—	8.6
C169	13.3	16.4	19.0	—
C171	15.7	17.0	22.4	—
C173	17.3	21.5	20.8	13.9
C195	16.4	19.5	21.3	13.9
C197	16.5	18.9	24.3	15.3
C206	14.6	16.7	20.7	12.9

“—” corresponds assays without inhibition zone.

The samples were treated with proteolytic enzymes and no inhibition zones was observed against *S. aureus* CECT-237, suggesting that the antimicrobial effect is provided by BLIS as suggested by previous works^63–66^.

**Figure 2:**
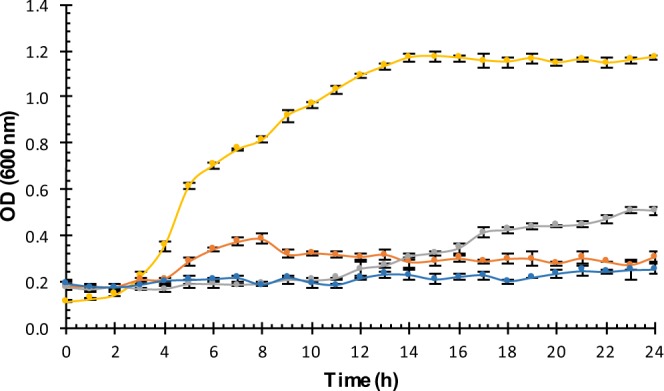
Antimicrobial activity of CFS-B extracted from culture broth of C43 (gray markers); C173 (blue markers); and C195 (orange markers) against *Salmonella* Heidelberg IOC 969/17 (yellow markers). Error bars correspond to standard deviations over three replicates.

Considering that neutralized samples from C43, C173 e C195 did not produced antimicrobial activities against *Salmonella* species, untreated samples (CFS-B) were evaluated against *S*. Heidelberg IOC 969/17. As can be observed in Fig. 2, all three samples demonstrated a satisfactory growth inhibition of *S*. Heidelberg IOC 969/17, especially the CFS-B extracted from C173 and C195. These results suggest that the antimicrobial activity produced by C43, C173 and C195 isolates against *S*. Heidelberg IOC 969/17 is not provided by BLIS, but by organic acids produced during their growth. When the culture media without bacterial growth was used as a control, *S*. Heidelberg inhibition was not observed (data not shown).

The use of such substances to combat *Salmonella* species in poultry products or products is very well documented^67–70^. The antimicrobial activity of organic acids can be explained because these substances can pass through the membranes of the target cells which will be dissociated in the more alkaline interior, acidifying the cell cytoplasm^70^. Many authors have shown that organic acids produced by probiotic LAB have antimicrobial effects and can be used as feed additive^71–74^. Furthermore, it has been shown that LAB can produce short and medium chain fatty acids (SCFA and MCFA) and other metabolites in the gastrointestinal tract of poulty that possess antagonism properties^67^ against different *Salmonella* species and on other microorganisms found in the avian microbiome^67,70,73^. Our results concur with these previous studies and suggest that our isolates could be useful to be used as supplements for poultry feed.

### Screening for vitamin producing isolates

In poultry nutrition, the deficiency of B-complex vitamins, especially riboflavin and folates can directly affect the health and well-being of birds.

Riboflavin are present in numerous co-enzymes that are involved in oxidation-reduction reactions for cell respiration^75^. When there is riboflavin deficiency in poultry, their growth is reduced caused by the loss of appetite and the appearance of diarrhea is very common^75^. Frequently, riboflavin deficient chicks cannot walk due to leg or curled-toe paralysis^76^. In addition, riboflavin deficiency in hens has been shown to lower egg production, cause the death of embryos, and increase the fat content in their livers^75,77^.

Folate also acts as a co- enzyme by accepting or donating single-carbon in amino acids and nucleic acids synthesis^31^. This vitamin is essential for purine and methyl groups synthesis which is why folates are essential for cell multiplication^75^. Folate deficiencies can reduce growth, cause megaloblastic anemia, produce poor feathering, achromat of feathers in colored poultry, and chondrodystrophy^31,75^.

Riboflavin and folate producing LAB can be an interesting option to supplement the diets of animal diets since they could release the vitamin in their feed or produce these essential nutrients in the gastrointestinal tract (although it is still unknown how much vitamins can be produce in situ).

In our study, among the 35 isolates selected in the previously step, those displaying the largest inhibition zones against the bioindicator strains were used to study their ability to produce folates and/or riboflavin. Eight isolates were selected, as means C43, C81, C83, C173, C195, C197 and C206. Posteriorly, these isolates were cultured in media culture broths free from folic acid and riboflavin and after 7 subcultures, from 8 isolates, 3 of them (C43, C173, and C195) grew well in the riboflavin free-culture medium and 5 isolates (C43, C81, C83, C173 C195) presented a good growth in the folic acid free-culture medium. Three of these 8 isolates (C43, C173, and C195) showed good growth in both culture media, suggesting riboflavin and folates are produced simultaneously.

As can be observed in Table 2, the isolates C195 and C173 showed the higher folate production (595 and 58 ng/mL respectively).

**Table 2:** Total folic acid concentration produced by the isolates selected on the step of screening of acid folic LAB producers. Error bars correspond to standard deviations over three replicates.

Name of isolates	Folic acid concentration (ng/mL)
C43	11.3 (±0.34)
C81	11.3 (±0.10)
C83	6.7 (±0.88)
C173	58.0 (±2.51)
C195	595.5 (±1.01)

The total riboflavin production by the 3 isolates are similarly, resulting in 230.0 ng/L (±0.04) by C43; 223.3 ng/L (±0.02) by C173; and 175.0 ng/mL (±0.04) by C195.

Many researchers stated the folate and riboflavin production by lactic acid bacteria^30,78–81^, but few have already reported the simultaneous production of both B-complex vitamins by a single strain, which represents an important advantages of our findings, especially for the poultry industries, since the LAB producing B-complex vitamins are mainly focused in human consumptions, instead of animals nutrition^82^.

According to the document published by National Research Council in 1994, the daily intake requirement of folic acid and riboflavin is about 1 and 5 mg per kg of diet, respectively^83^. The daily feed consumption varies considerably between gender and also the poultry specification, *i.e*. laying hens and broiler chicken, for examples. For broiler chicken, a 1-day old male chicken consumes about 13 g of feed^84^. When the same chicken reaches 45 days of life, its daily consumption is of approximately 186 g of feed. The same estimative can be done about water intake. A 1-week broiler chicken can drinks about 40 mL of water per day, which increase 5 times when this chicken is 3-weeks old^84^.

Feed for poultry are formulated to make sure that they contain more than enough micronutrients (vitamins and minerals) to avoid deficiencies and take into account the amounts that are lost during their processing, storage and transport^75^. In fact, this is sometimes questioned as being too costly^84^. Thus, including our isolates in commercial poultry nutrition can be an economic alternative. Another advantage is associated to the fact that they could be more stable to storage conditions, one of the major causes of loss of vitamins bioavailability^75^.

### Identification of the selected isolates by MALDI-TOFMS Biotyper

Considering the best performance of the isolates C43, C173 and C195, the genus and species of such microorganisms were identified by MALDI-TOFMS Biotyper that identifies microorganisms by using protein profiles of microorganisms and comparison with known patterns^47,85^. This technique is fast, economic and is a reliable alternative to conventional 16 s rRNA PCR amplification and sequencing to identify microorganisms^85,86^.

The isolate C43 was identified as *E. faecium*, whereas C173 and C195 were both identified as being strains of *L. lactis* subsp. *lactis*.

The presence and the isolation of *E. faecium* from chicken microbiota is very well documented in the literature^87–89^, but this is not the case for *L. lactis* strains. This species is normally found in dairy, vegetable, sausages, raw pork, vacuum-packed seafood^90^ and from chicken cecum^91^.

According to the Food and Agriculture Organization^92^, both species have been used as probiotics in poultry diets. *E. faecium* has shown to be able to increased growth performance, improved intestinal morphology, and possess antagonism effects against important pathogens in poultry production^93,94^. Its usage in poultry nutrition is so widely defunded that are many commercial feed composed with *E. faecium*, for example PoultryStar^®^, Protexin^®^, and Biomin^92^.

Although the use of *L. lactis* in poultry is not very common, Maiorano *et al*.^95^ demonstrated increased feed conversion ratio (FCR) when a symbiotic mixture of *L. lactis* ssp. *cremoris* IBB SC1 and raffinose were injected in chickens’ eggs in order to evaluate its effect on productivity, the quality of the meat produced, and on the incidence of pectoral muscle pathologies in broiler chickens. On the other hand, Pruszynska-Oszmalek *et al*.^96^ reported that of *L. lactis* subsp. *lactis* added with a prebiotic mixture of galacto-oligosaccharide and inulin did not result in improved FCR, but significantly increased final body weight of the chickens tested. Another interesting approach is a lactococcal vaccine proposed by Reese and collaborators^97^. In this study, the author demonstrated the protected effect of the vaccinated birds against the avian influenza virus.

As can be observed, both species, i.e. *E. faecium* and *L. lactis* subsp. *lactis* have being proven their benefits to chickens health when administrated as probiotic bacteria.

### Evaluating the probiotic potential of the selected isolates

**Figure 3:**
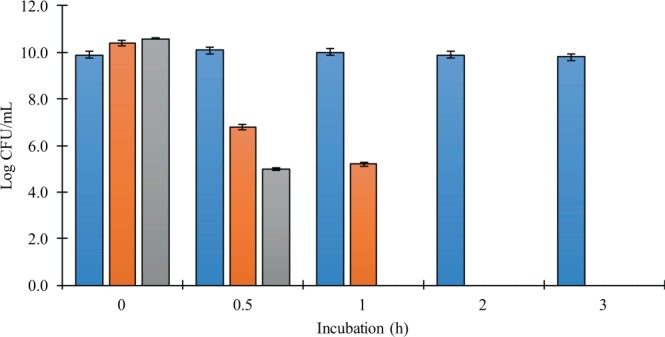
Viability of the isolates C43 (gray bars), C173 (blue bars), and C195 (orange bars) after 0, 0.5, 1, 2, and 3 h incubation under low pH (2.5). Error bars correspond to standard deviations over three replicates.

In order for orally ingested bacteria to be able to exert a beneficial effect on their host, they must survive the passage through the stomach and the presence of bile salts in the intestinal lumen and arrive alive in the large intestines^98^. As can be observed in Fig. 3, except the C43, the isolates C173 and C195 showed significantly loss of viability in low pH conditions; however, their encapsulation or addition in a food matrix could increase their survival^50^.

Although bovine bile is normally used to evaluate the tolerance of bacteria, bile salts from pork are more similar to that produced by humans^99^, justifying its using in the present study.

**Figure 4:**
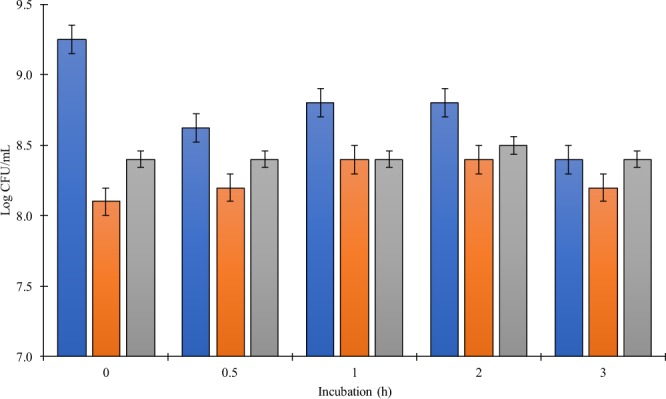
Survival of the isolates C43 (gray bars), C173 (blue bars), and C195 (orange bars) after exposition 0.5% bile salts for 0, 0.5, 1, 2, and 3 h incubation. Error bars correspond to standard deviations over three replicates.

Overall, bile salts at 0.5% concentration did not affect the survival of the isolates C43, C173 and C195 (Fig. 4)

Bile salt concentrations are crucial as a defense mechanisms by inhibiting the survival of microorganisms^100^. The resistance to bile salts is this critical for probiotic strains selection in order for them to arrive in a live form after its passage in the small intetines^49^.

#### Hemolytic activity and antibiotic resistance

None of the selected isolates demonstrated β or α-hemolytic activities, essential for the possible use of the strains^101^.

Because of the increasing problems concerning antibiotic resistance, it is important that probiotics that are to be used in poultry feed additive not be reservoirs for antibiotic resistance genes^102,103^.

According to zones diameters breakpoints established by Charteris *et al*.^54^, all isolates were sensitive to the studied antibiotics except for *L. lactis* subsp. *lactis* C173, which showed resistant to vancomycin. This result is similar to those of other studies^50,82,98^. Although it was previously reported certain strains of *L. casei, L. rhamnosus, L. plantarum*, pediococci and *Leuconostoc* spp. are resistant to vancomycin, this property is of much concern because this antibiotic is is one of the last antibiotics that is useful in infections caused by multidrug-resistant pathogens^104,105^. Based in the study reported by Temmerman *et al*.^106^, it would be appropriate to use other methods to confirm the presence of vancomycin resistance in *L. lactis* subsp. *lactis* C173, like Minimal Inhibitory Concentration (MIC) or PCR assay, which will be considered in a future study.

#### Coexistence test

With the aim of obtaining multi-strain probiotics, and due to their antimicrobial properties, strain compatibility must be evaluated^107^.

**Figure 5:**
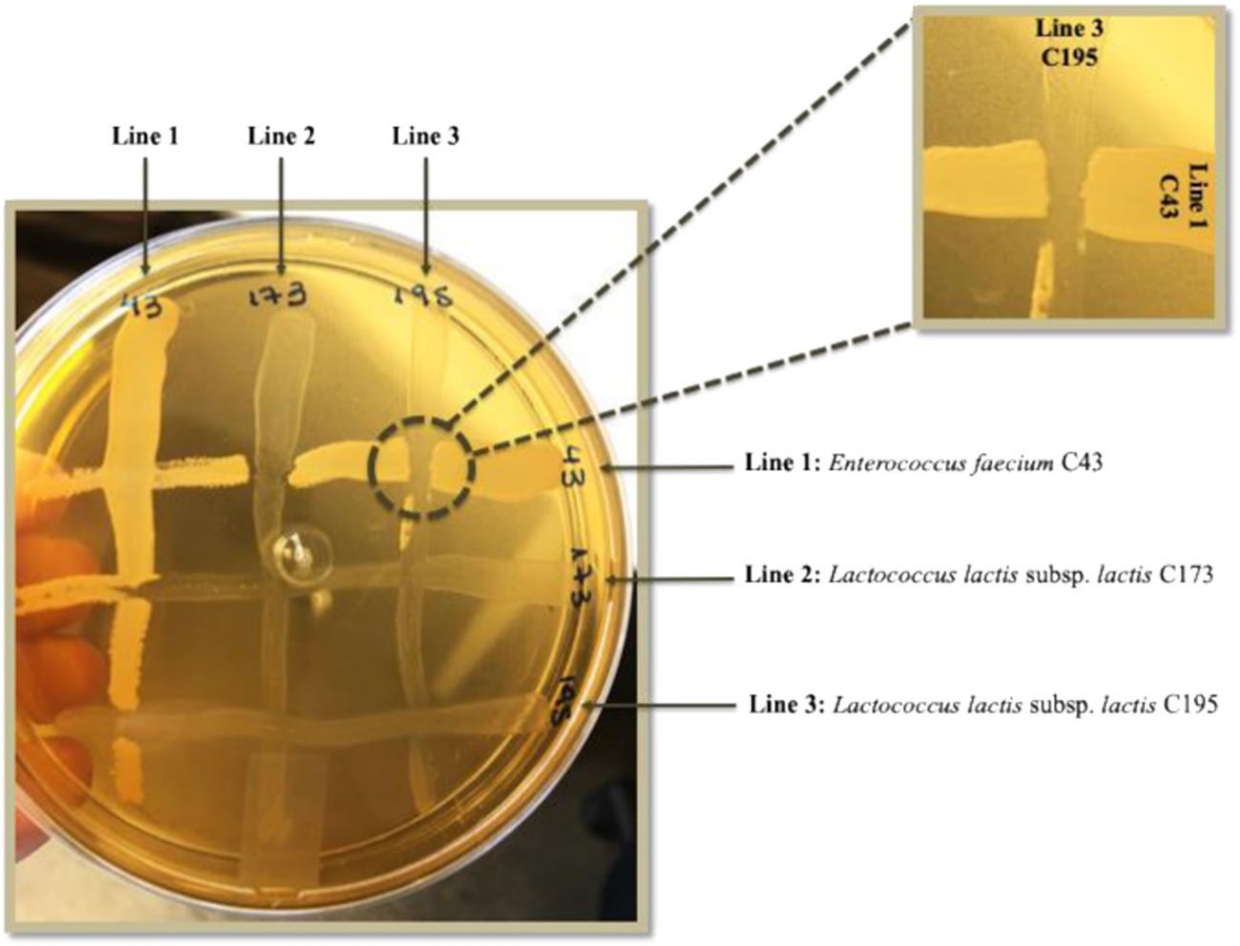
Isolates compatibility demonstrated by coexistence test. The cross-streaked assay reveled the antagonism effect of *Lactococcus lactis* subsp. *lactis* C173 (Lines 2) and *Lactococcus lactis* subsp. *lactis* C195 (Lines 3) against *Enterococcus faecium* C43 (Lines 1). The intersection between the lines 1 and 3 is extended in the figure inset.

As can be observed in Fig. 5, the isolate *L. lactis* subsp. *lactis* C173 and *L. lactis* subsp. *lactis* C195 reveled their antagonistic effect against *E. faecium* C43. Based on this data and assuming that this effect could also occur *in vivo*, the *E. faecium* C43 were excluded from the following tests.

#### Hydrophobicity property and adhesion of the selected isolates to Caco-2 cells

In order to evaluate their potential action in the gastrointestinal tract, adhesion to human colon carcinoma and the evaluation of hydrophobicity must be performed^107^. Hydrophobicity has been associated with bacterial adhesion properties^108^ reason for which the isolates’ adhesion to toluene was measured. The *L. lactis* subsp. *lactis* C173 showed a moderate hydrophobicity (44%), while *L. lactis* subsp. *lactis* C195 hydrophilic surface (9.2%).

**Figure 6:**
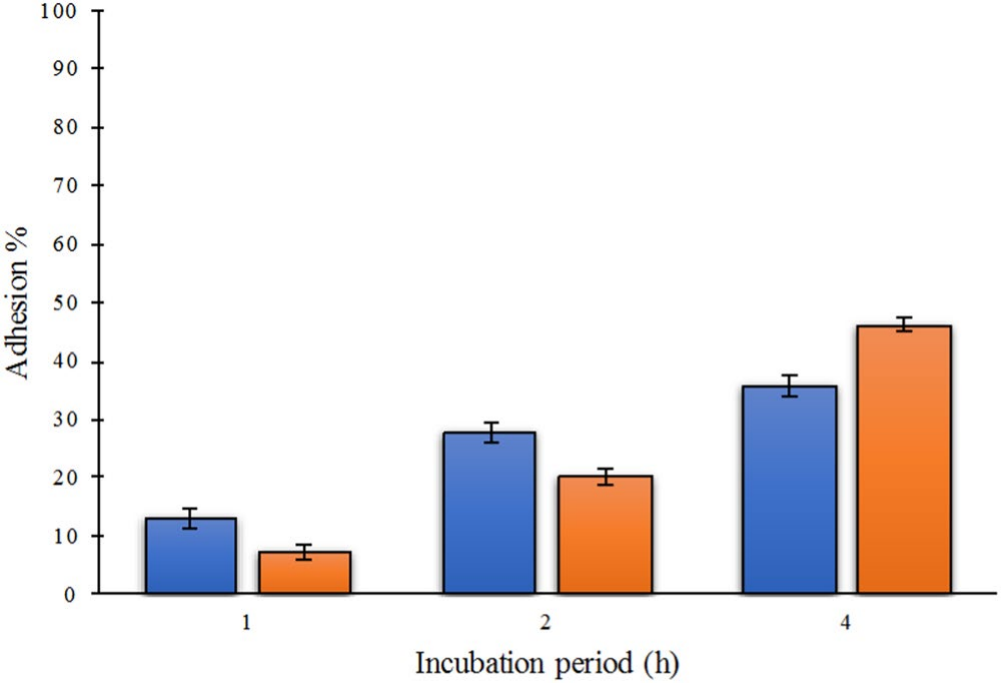
Adhesion (%) of the isolates *Lactococcus lactis* subsp. *lactis* C173 (blue bars) and *Lactococcus lactis* subsp. *lactis* C195 (orange bars) to Caco-2 cells after 1, 2 and 4 h incubation. Error bars correspond to standard deviations over three full biological replicates.

Adherence to Caco-2 assay showed that both isolates presented high value of adhesion to this cells (Fig. 6). For *L. lactis* subsp. *lactis* C173, after 1, 2 and 4 h incubation, the bacterial solution presented values of adhesion of 13.2, 27.9 and 35.6%, respectively. *L. lactis* subsp. *lactis* C195 in turn, resulted in adhesion values of 7.3, 20.3 and 46.3% after 1, 2 and 4 h incubation, respectively. According to Gharbi *et al*.^98^, Laiño *et al*.^109^ and Jensen *et al*.^57^ an adhesion rate of 11% is considered a high adhesion capacity. In this study, it is clear that a longer period of incubation results higher adhesion percentages, but independently of this incubation time, both cells showed strong adhesion with Caco-2 cells. This high adhesion percentage suggests that these strains are likely to colonize the GI tract. It is important that probiotics adhere to the intestinal epithelium in order to facilitate their colonization and prevent pathogen binding^110^.

As mentioned before, bacterial cell hydrophobicity is frequently associated to the adherence to the human epithelium cells; however there is conflicting reports on this direct association^98,111^. Our findings are in agreement with these latter authors that there is no direct association between hydrophobicity and the binding habilities of our isolates.

## Conclusion

From 314 isolates from chicken cecum, the isolates labeled as C43, C175 and C195 demonstrated antagonistic effect towards many pathogens. Using proteolytic enzymes, it was demonstrated that the antimicrobial effect against *Listeria* species, *C. piscicola* and *S. aureus* was caused by BLIS and by organic acids against *S*. Heridelberg. The isolates, identified as *E. faecium* and *L lactis* subsp. *lactis*, also produced the B-complex vitamins folate and riboflavin, which is very interesting considering the diseases related to the lack of these nutrients in the feeding of birds. After *in vitro* probiotic assays, the *L. lactis* subsp. *lactis* strains C173 and C195 demonstrated high potential to be used as probiotic in poultry feed being a useful alternative to replace antibiotics in poultry husbandry. In order to demonstrate this potential, in-vivo trials will be required and are currently underway.
